# Supplementary material for: Defatted Seed Residue of Cucumis Melo as a Novel, Renewable and Green Biosorbent for Removal of Selected Heavy Metals from Wastewater: Kinetic and Isothermal Study
Source: Molecules. 2022 Oct 7;27(19):6671. doi: 10.3390/molecules27196671 (PMC9573229; doi:10.3390/molecules27196671)
Supplement: Supplementary file 1 [file molecules-27-06671-s001.zip › molecules-1917272-supplementary.pdf]

SUPPLEMENTARY MATERIAL

FOR

“Defatted Seed Residue of *Cucumis melo* as a Novel, Renewable and Green Biosorbentfor Removal of Selected Heavy Metals from Wastewater: Kinetic and Isothermal Study”

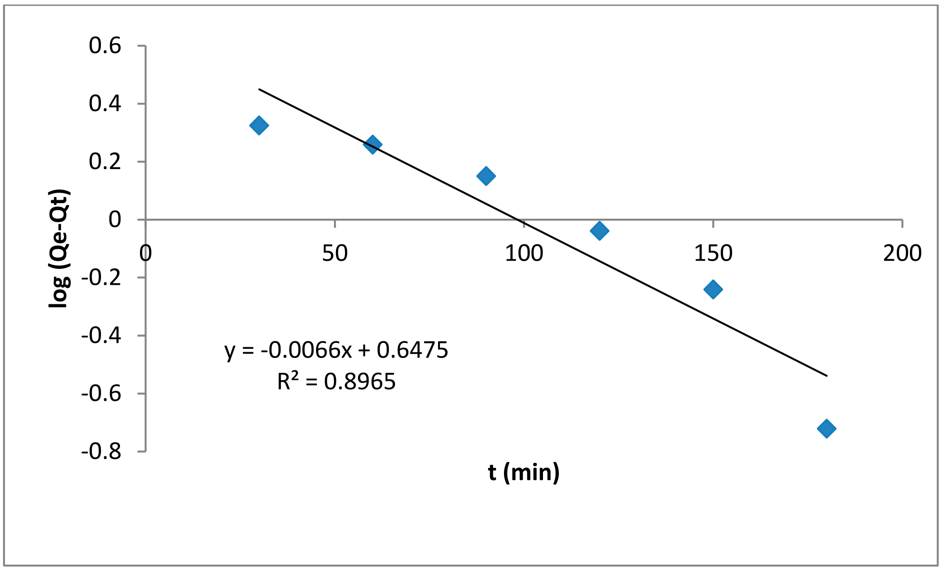

Figure S1. **Plot of log (Qe-Qt) Vs. time for Pseudo-first order kinetic model (Linear Form), for Pb**

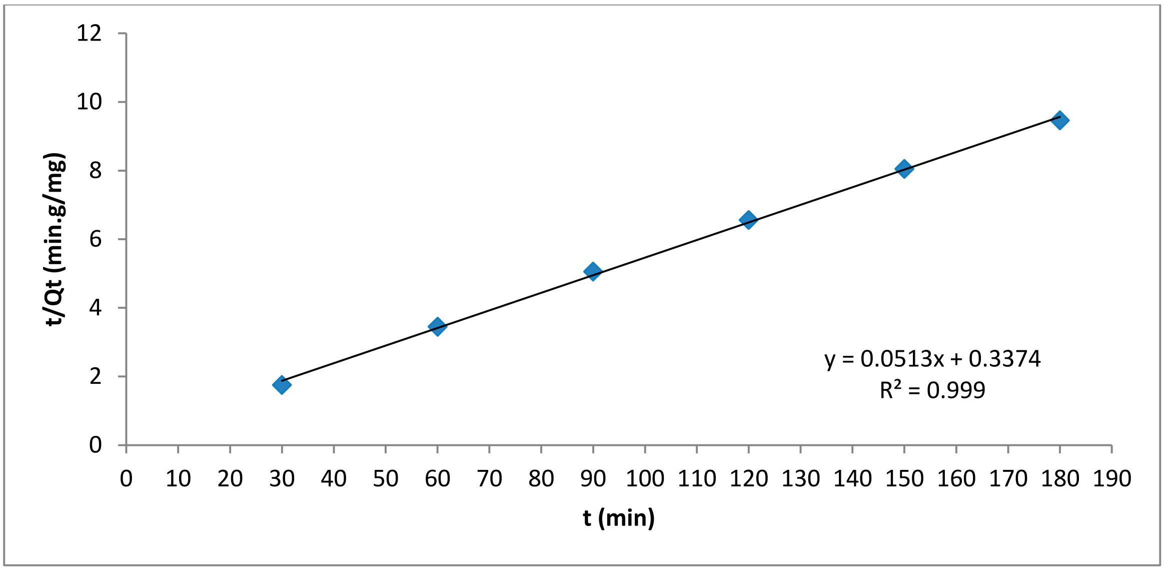

Figure S2. **Plot of t/Q_t Vs time for Pseudo-second order kinetic model (Type 1), for Pb**

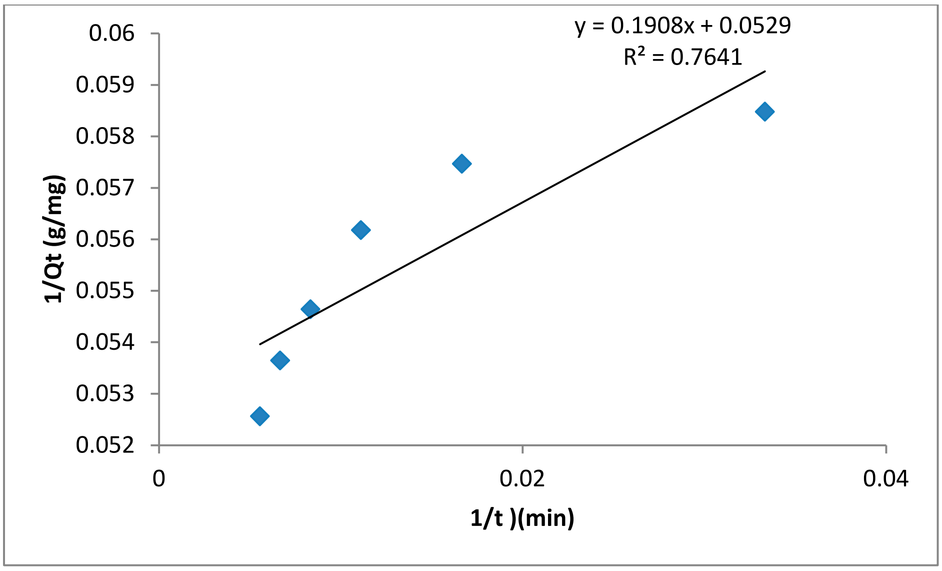

Figure S3. **Plot of 1/Q_t Vs 1/t for Pseudo-second order kinetic model (Type 2), for Pb**

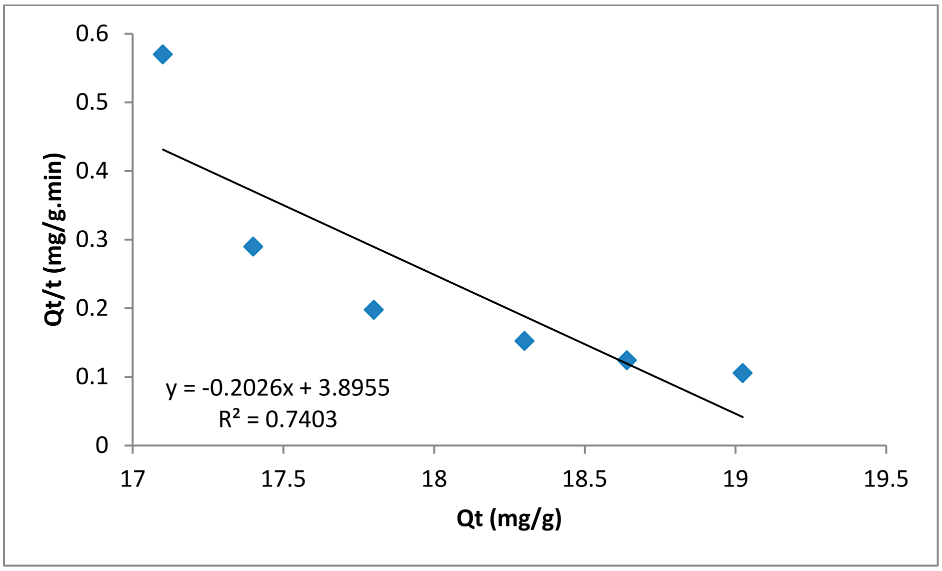

Figure S4. **Plot of Q_t/t Vs. Q_t for Pseudo-second order kinetic model (Type 3), for Pb**

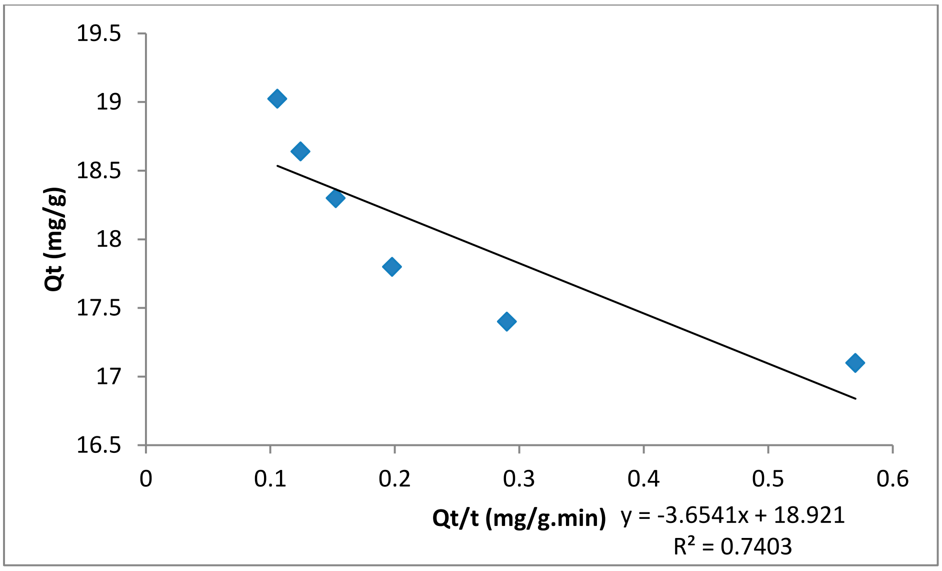

Figure S5. **Plot of Q_t Vs. Q_t/t for Pseudo-second order kinetic model (Type 4), for Pb**

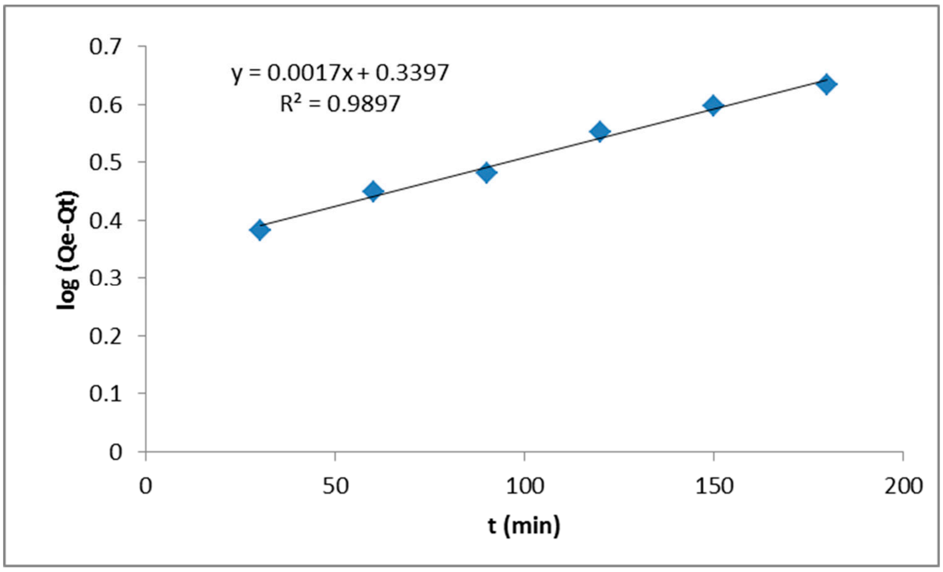

Figure S6. **Plot of log (Qe-Qt) Vs. time for Pseudo-first order kinetic model (Linear Form), for Cr**

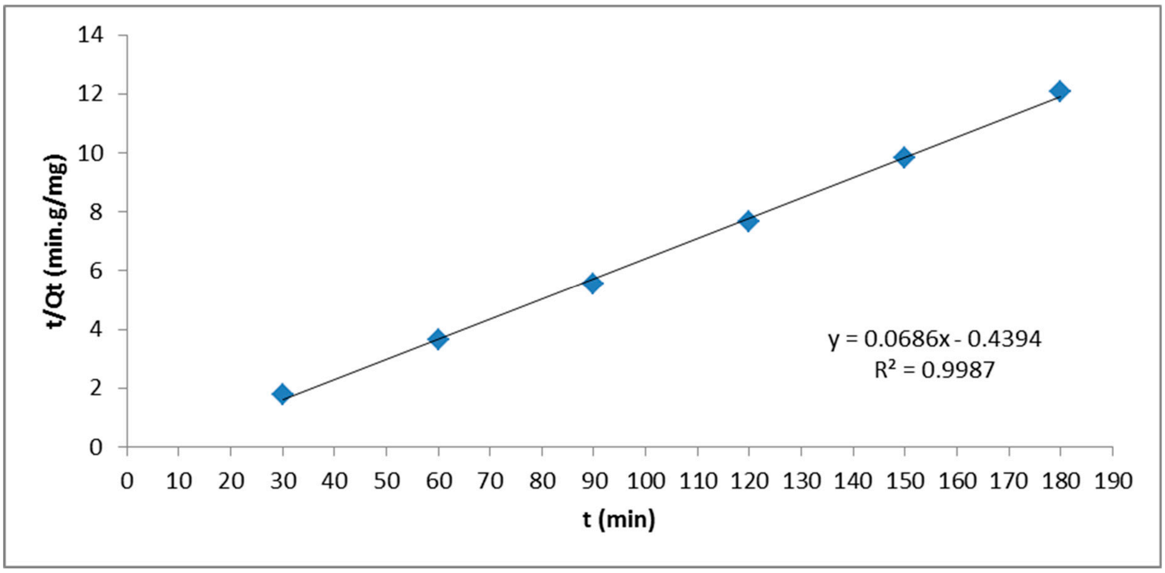

Figure S7. **Plot of t/Q_t Vs time for Pseudo-second order kinetic model (Type 1), for Cr**

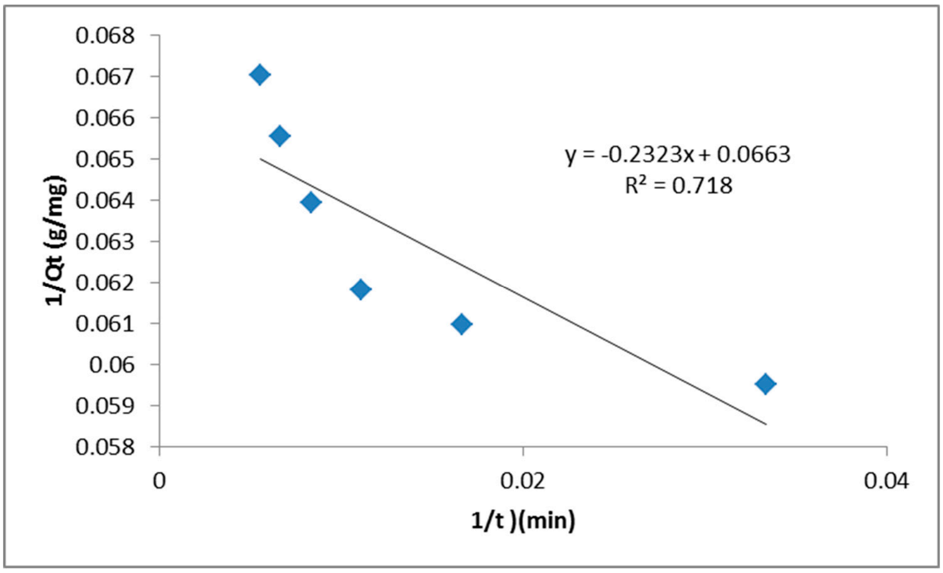

Figure S8. **Plot of $1/Q_t$ Vs 1/t for Pseudo-second order kinetic model (Type 2), for Cr**

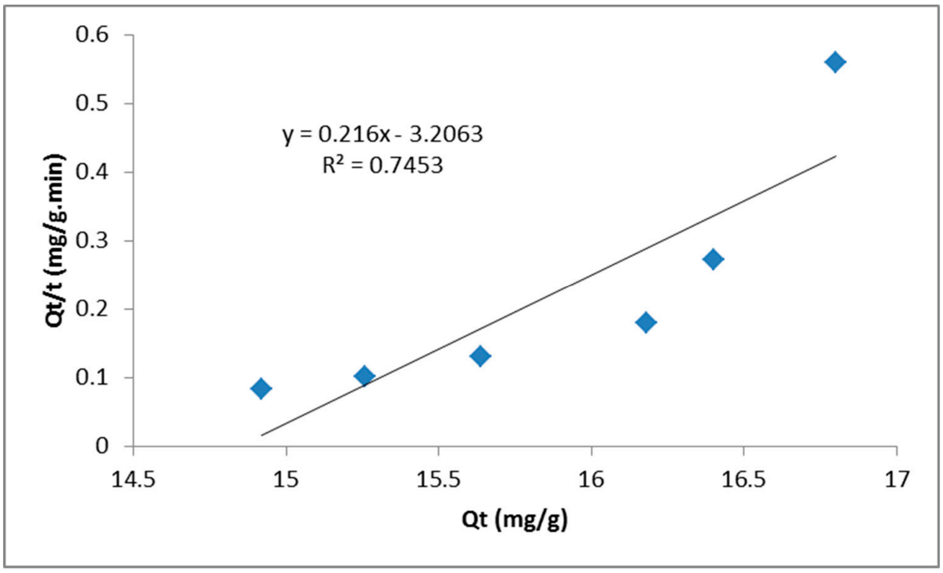

Figure S9. **Plot of Q_t/t Vs. Q_t for Pseudo-second order kinetic model (Type 3), for Cr**

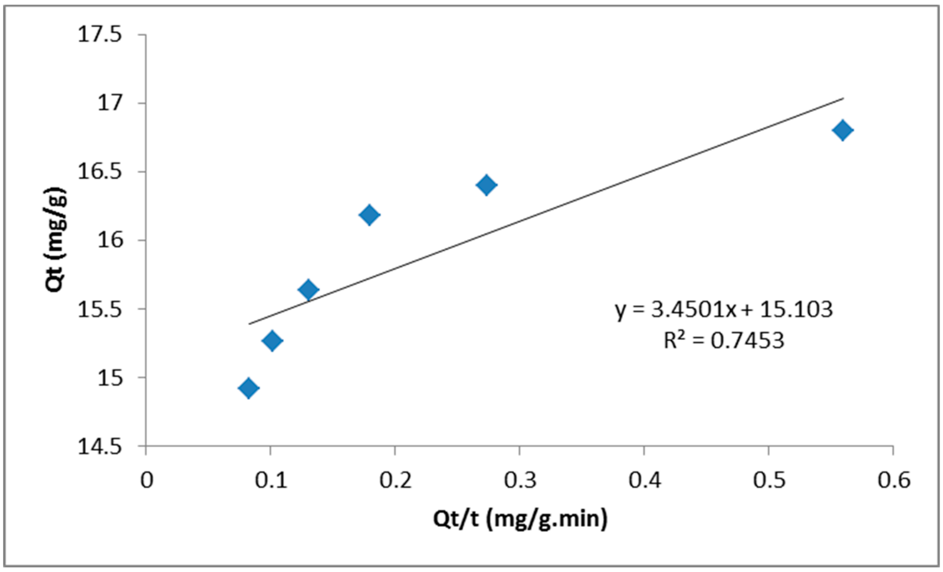

Figure S10. Plot of Q_t Vs. Q_t/t for Pseudo-second order kinetic model (Type 4), for Cr

Table S1. Pseudo-First-Order and Second-Order Kinetic Models Applied on Present Work (biosorption of Pb and Cr on defatted seed residue of *C. melo*

**Pseudo-Second-Order Model Form**		**Equation**	**Plot**	**Parameters**
**Linear Forms**	Type 1	$\frac{t}{Q_t} = \frac{1}{k_2 Q_e^2} + (\frac{1}{Q_e})\ t$	t/Q_t vs. t	Q_e = 1/Slope<br>h = 1/Intercept<br>k = $(\text{slope})^2$/Intercept
	Type2	$\frac{1}{Q_t} = (\frac{1}{k_2 Q_e^2})\frac{1}{t} + (\frac{1}{Q_e})$	$1/Q_t$ vs. 1/t	Q_e = 1/Intercept<br>h = 1/Slope<br>k = $(\text{Intercept})^2$/Slop
	Type 3	$\frac{Q_t}{t} = kQ_e^2 - kQ_e Q_t$	Q_t/t vs. Q_t	Q_e = -Intercept/Slop<br>h = Intercept<br>k = $(\text{Slop})^2$/Intercept
	Type 4	$Q_t = Q_e - (\frac{1}{kQ_e})\frac{Q_t}{t}$	Q_t vs. Q_t/t	Q_e = Intercept<br>h = -Intercept/Slop<br>k = -1/(Intercept x Slop)
**Pseudo-Second-Order Non-Linear Form**		$Q_t = \frac{k_2 Q_e^2 t}{1 + k_2 Q_e t}$	$1/Q_t$ vs. 1/t	Q_e = 1/Intercept<br>h = 1/Slope<br>k = $(\text{Intercept})^2$/Slop
**Pseudo-First-Order Lineal Form**		$\ln(Q_e - Q_t) = \ln(Q_e) - k_1 t$	$\ln(Q_e - Q_t)$vs. t	k = Slop

**Pseudo-First-Order Non-Lineal Form**	$Q_t = Q_e(1-e^{-k_1t})$	$\ln(Q_e-Q_t)$vs. t	k= Slop
